# Mental Health Service User and Worker Experiences of Psychosocial Support Via Telehealth Through the COVID-19 Pandemic: Qualitative Study

**DOI:** 10.2196/29671

**Published:** 2021-08-12

**Authors:** Annie Venville, Sarah O'Connor, Hannah Roeschlein, Priscilla Ennals, Grace McLoughlan, Neil Thomas

**Affiliations:** 1 Social Work College of Health and Biomedicine Victoria University Footscray Australia; 2 Neami National Melbourne Australia; 3 Centre for Mental Health Swinburne University of Technology Melbourne Australia

**Keywords:** telehealth, mental health, psychosocial support, COVID-19, service user, workers, qualitative, e-mental health, support, telemedicine, intervention, user experience

## Abstract

**Background:**

During the COVID-19 pandemic, we saw telehealth rapidly become the primary way to receive mental health care. International research has validated many of the benefits and challenges of telehealth known beforehand for specific population groups. However, if telehealth is to assume prominence in future mental health service delivery, greater understanding of its capacity to be used to provide psychosocial support to people with complex and enduring mental health conditions is needed.

**Objective:**

We focused on an Australian community-managed provider of psychosocial intervention and support. We aimed to understand service user and worker experiences of psychosocial support via telehealth throughout the COVID-19 pandemic.

**Methods:**

This study was jointly developed and conducted by people with lived experience of mental ill health or distress, mental health service providers, and university-based researchers. Semistructured interviews were conducted between August and November 2020 and explored participant experiences of receiving or providing psychosocial support via telehealth, including telephone, text, and videoconferencing. Qualitative data were analyzed thematically; quantitative data were collated and analyzed using descriptive statistics.

**Results:**

Service users (n=20) and workers (n=8) completed individual interviews via telephone or videoconferencing platform. Service users received psychosocial support services by telephone (12/20, 60%), by videoconferencing (6/20, 30%), and by both telephone and videoconferencing (2/20, 10%). Of note, 55% (11/20) of service user participants stated a future preference for in-person psychosocial support services, 30% (6/20) preferred to receive a mixture of in-person and telehealth, and 15% (3/20) elected telehealth only. Two meta-themes emerged as integral to worker and service user experience of telehealth during the pandemic: (1) creating safety and comfort and (2) a whole new way of working. The first meta-theme comprises subthemes relating to a sense of safety and comfort while using telehealth; including trusting in the relationship and having and exercising choice and control. The second meta-theme contains subthemes reflecting key challenges and opportunities associated with the shift from in-person psychosocial support to telehealth.

**Conclusions:**

Overall, our findings highlighted that most service users experienced telehealth positively, but this was dependent on them continuing to get the support they needed in a way that was safe and comfortable. While access difficulties of a subgroup of service users should not be ignored, most service users and workers were able to adapt to telehealth by focusing on maintaining the relationship and using choice and flexibility to maintain service delivery. Although most research participants expressed a preference for a return to in-person psychosocial support or hybrid in-person and telehealth models, there was a general recognition that intentional use of telehealth could contribute to flexible and responsive service delivery. Challenges to telehealth provision of psychosocial support identified in this study are yet to be fully understood.

## Introduction

COVID-19 has resulted in a dramatic increase in the use of telehealth for mental health treatment, intervention, and support. Although there had been incremental growth in the use of telehealth modalities such as videoconferencing and telephone prior to the pandemic, it had largely been seen as a substitute for in-person service provision, particularly for people unable to travel or located in remote or underserved regions; evidence showed that videoconferencing and telephone delivery was feasible with high satisfaction and equivalent outcomes, and in some cases, could reduce discomfort associated with in-person interactions [1-6]. Effective group facilitation via videoconference [7-9], and decreased feelings of anxiety and isolation in group videoconferencing settings, even for those with limited digital literacy [10], had also been reported.

Some of the most commonly cited barriers to implementing telehealth services before the pandemic included reduced opportunities for nonverbal cues and eye contact [8], reduced worker confidence in rapport-building and limited worker buy-in [11], technical issues (including poor audio quality, time lag, problems downloading software, and slow internet connection) [10]; inequitable access to internet, and reduced rapport between participants and workers [7-9]. Challenges notwithstanding, in the face of widespread international service disruption wrought by COVID-19, telehealth rapidly became, for many, the primary way to receive mental health care.

During the pandemic, extensive reporting of the benefits of telehealth consultations [12] for mental health assessment, treatment, interventions, and ongoing support largely validated the prepandemic experiences of psychiatrist- or psychologist-based services and psychoeducational programs (eg, chronic disease management, therapeutic support for anxiety and depression, and support groups). However, there has been less direct study of the impact on psychosocial support delivered to people with moderate to severe mental health problems. Psychosocial support aims to assist people make sense of what is going on for them, explore possibilities, and manage daily tasks in their natural environments—the effectiveness of which is typically reliant on strong relationships between service users and workers. These types of interventions frequently involve the provision of practical support and “walking alongside” people as they engage in work, home, and community life. The pandemic further tested the mental health of many people by creating a situation that restricted people from natural support, community connections, and the routines and rhythms of daily life that contribute to the well-being of individuals, communities, and society [13].

This project explored the provision of psychosocial support via telehealth for people with complex mental health conditions. The aims of this study were twofold—first, to understand the service user and workers experience of provision of psychosocial support via telehealth through the COVID-19 pandemic, and second, to use findings to inform planning for the incorporation of telehealth into future service delivery.

## Methods

### Study Setting

The study focus was an Australian community-managed provider of psychosocial intervention and support called Neami National. Neami community support workers provide tailored psychosocial interventions focused on supporting adults with moderate to severe mental health problems such as psychotic disorders and severe mood disorders, to live well in the community or navigate acute distress or ill health. Neami assists over 9000 people each year to work toward goals that may include community participation, managing daily tasks, undertaking work or study, finding housing, and strengthening social connections. These psychosocial supports and interventions are commissioned by federal or state governments or local health districts and are regularly described by service users as central to sustaining their mental health and well-being, increasing their ability to cope with the ups and downs of life, and reducing their need to access more intensive, clinical or acute mental health services.

Prior to the pandemic, Neami predominantly provided psychosocial supports for individuals or groups in face-to-face settings with infrequent telehealth offerings. During the COVID-19 response period, government lockdown restrictions affected Neami service provision across Australia, and almost overnight, in-person psychosocial support transitioned to telehealth (videoconferencing, text or instant messaging, and telephone). Nationally, staff continued to provide a range of service offerings including group and individual work via telehealth; however, the more practical face-to-face supports could not be provided in their usual way. This study took place between August and November 2020, during which Victoria was at the epicenter of Australia’s COVID-19 second wave crisis; Victoria experienced the most severe and prolonged lockdown in Australia (15 weeks). During this time, other Australian states and territories experienced fewer restrictions; however, telehealth was widely offered. Neami sought to learn from pandemic-forced changes to service delivery by understanding worker and service user experiences of these different ways of receiving psychosocial support and identifying the barriers, enablers, and opportunities for telehealth in psychosocial- or recovery-focused practice.

### Design

We designed a qualitative exploratory methodology that reflected an interpretive approach [14] to understand service user and worker experience of psychosocial support via telehealth through the COVID-19 pandemic. The research was jointly developed and conducted by people with lived experience of mental ill health or distress, Neami staff, and university-based researchers.

### Participants

A combination of purposive, convenience, and snowball sampling was used to recruit 2 participant groups—service users and workers—from a range of Neami sites across Australia. The study was promoted to service users (1) via information posted on a digital platform enabling direct contact with the research team, and (2) through conversation with Neami workers, who obtained permission to pass on contact details to the researchers. Workers were informed of the study through internal Neami channels and team meetings. Workers with experiences of service users being challenged by telehealth or who had become disconnected because of telehealth were intentionally sought out. Prospective participants who expressed interest in the research received detailed information about the study via an email or telephone call from a member of the research team (AV). A second contact was made with prospective participants to gain consent to participate and arrange a preferred time and modality for the interview—phone, videoconference, or a hard copy of the interview questions if they wished to respond in writing. Participants gave verbal consent at the commencement of the interview or signed and returned a written consent form prior to interview. The voluntary nature of participation was explicitly communicated, and people were advised that they could change their mind or withdraw at any time. They were also advised that their choice to not participate or to withdraw participation would not in any way affect their relationship with Neami, Neami workers, or the service they provide or receive.

### Data Collection

A semistructured interview guide was used with both service users and workers. Questions explored participant experiences of receiving or providing psychosocial support via telehealth platforms including telephone, text, and videoconferencing. We were particularly interested in learning about the challenges and opportunities telehealth support offered during a global pandemic, how it differed from the types of support people had received or provided in the past, and what role they saw it playing into their future. The interviews were conducted by 5 researchers (AV, PE, SO, HR, GM) and took place between August and November 2020. They ranged in length from 30 to 60 minutes and were recorded and transcribed verbatim. Service user participants received a Aus $40 (approximately US $29.45) gift voucher as a token of appreciation.

### Data Analysis

Interview data were analyzed thematically in keeping with the techniques described by Braun and Clark [15]. The process was inductive and began with 3 researchers (AV, HR, and SO) rereading the transcripts to increase familiarity with the data and noting ideas as preliminary codes. A discussion of these ideas with the whole research team then led to the generation of initial categories. Researchers (AV, HR, SO, and GM) returned to the transcripts and organized data relevant to each category. The categories were reviewed with the research team for input and feedback, and then, mapped to generate themes and subthemes. Themes were again reviewed, refined, and summarized by all researchers to ensure that they had clear parameters and fit with the coded extracts. Data extracts were checked for accuracy (SO, HR, AV). The study was approved by the University ethics committee (HRE20-115) and Neami.

## Results

Table 1 provides a worked sample of the theme development.

**Table 1:** Subtheme development.

Theme	Subthemes	Categories	Sample quotations
Creating safety and comfort	Trusting in the relationship	Trusting workers—feeling safe	“We did develop quite a good relationship, I would say. I feel quite comfortable being able to talk to her and, you know, let her know what's happening” [Li, Service use] “she's always been really good and dedicates a lot of time to me“ [Christine, Service user] “we all started off a bit clunky and then as confidence increased and as I think you said before, you know, part of that rapport building is just kind of being a bit - you know, the genuineness and if people can do that, then that encourages rapport and then that encourages trust, and then that encourages the connection. So, it's those things, isn't it? It's a package deal.” [Julia, Service user]
		Determination to keep service users engaged	“They just found it too hard which was really disappointing because it's something I was quite excited about and they just found it too hard to do and I guess being able to sort of explain or sort of help someone navigate that process, it's kind of a bit ironic that, you know, I'm trying to do that via telehealth, sort of teach them how to use the telehealth and it just didn't work.” [Liam, Worker]
		Equalizing power—mutual learning (and we are all in this pandemic together)	“I feel like they know more about the platforms than we do. So, I feel sometimes they've helped us out with using it than the other way around.” [Megan, Worker]

### Participant Characteristics

Participants included 20 service users and 8 workers. Detailed diagnostic information was not collected from individual service users; Neami supports people with enduring mental health challenges and diagnoses—typically psychotic disorders, mood disorders, or personality disorders. Half of service user participants (10/20, 50%) and 38% of worker participants (3/8) lived in Victoria, which was subject to a prolonged period of lockdown during 2020. Most service users (17/20, 85%) had not used telehealth services for psychosocial support prior to the pandemic. Service users ranged in age from 17 to 68 years, with 55% service user participants (11/20) under 40 years of age and 45% over 40 years of age (9/20). Of the 20 service user participants, 16 (75%) self-identified as female, 4 self-identified (25%) as male, and 0 (0%) self-identified as nonbinary. All service users (20/20, 100%) described English as the main language spoken at home; 1 service user self-identified as Aboriginal and/or Torres Strait Islander, 4 self-identified cultural backgrounds including Iranian, Lebanese and Malay/Chinese. Four service users disclosed health conditions that they felt affected their ability to engage with telehealth including mild hearing impairments, back injury, migraines, chronic pain, complex PTSD, sensory issues, ear issues, and fatigue. It was not possible for us to contact service users who had disengaged from mental health support during the pandemic and impressions of their experiences of telehealth relied upon reports from workers. Of the 8 worker participants, 5 self-identified as female, and 3 self-identified as male. All worked in direct psychosocial support roles, with duration of employment at the Neami ranging from 10 months to 6 years.

### Current and Preferred Use of Telehealth Services

During the study period, 60% (12/20) of service user participants received psychosocial support services from Neami by telephone, 30% of service user participants (6/20) received support services by videoconferencing, and 10% of service user participants (2/20) received support services by both telephone and videoconferencing. For those who chose to disclose information about other telehealth services with which they had engaged (for example medicine, psychiatry, psychology), 18% (3/17) received services via phone, 41% (7/17) via videoconferencing, and 41% (7/17) via a blend of approaches. Of note, 55% (11/20) of service user participants stated a future preference for in-person psychosocial support services, while 30% (6/20) preferred to receive a mixture of in-person and telehealth, and 15% (3/20) elected telehealth only.

### Key Themes

Analysis of interview data revealed 2 major themes as integral to worker and service user experience of telehealth during the pandemic: (1) creating safety and comfort, and (2) a whole new way of working (Table 2).

**Table 2:** Themes, subthemes, and categories.

Themes and subthemes			Categories
**Creating safety and comfort**			
	Trusting in the relationship	Trusting workers—feeling safe Determination to keep service users engaged Equalizing power—mutual learning (and we are all in this pandemic together)	
	Matching service offering with service user need	Getting what I need (or not) Not getting practical support Recognizing and responding to the nature of distress The pros and cons of different platforms for different needs	
	Having and exercising choice and control	Options made explicit; choices offered Choices in platforms Choices in ways and timing of engagement Choices in content/focus	
	Spaces and strategies that enable privacy and safety	Private spaces to talk freely The value of physical therapeutic spaces Flexibility required for privacy and safety Digital security not really a concern Having time creates a sense of safety	
**A whole new way of working**			
	Doing things differently	Following the rules or making up the rules as we go Trying things out—Learning as we go and “winging it” Shifting assumptions about “good practice” Service users and workers showing flexibility	
	Good practice in telehealth takes time, effort and organizational support	Building skills and confidence Developing and adjusting to new ways of working Resourcing workers Resourcing service users	

### Creating Safety and Comfort

#### Trusting in the Relationship

Trust was a feature of strong and safe worker and service user relationships. This, alongside worker skills and attributes, positively influenced service users’ feelings of safety and comfort with telehealth. Service users expressed appreciation for workers showing patience and focus, taking the time to listen, being respectful and understanding, and adapting to reduced visual cues. All but one service user felt that telehealth sessions benefited from having a preexisting relationship with their Neami worker; this was also the case for telehealth sessions with their general practitioner, psychiatrist, or other health professional. For many service users, their sense of safety and comfort with telehealth increased with a developing therapeutic relationship, time, and practice:

Yeah, it was hard first, like our first few sessions because I was like, oh, new person again… and I was like, ”I'm scared I'm going to have to retell everything and be like kind of opening up trauma again.“ It wasn't like that at all. That was just my thoughts racing but our first session on the phone was like - she was very just like, ”Get to know each other, don't really talk about the mental health side of it yet.“ Then our second session was more comfortable. I knew her name, she knows me, and then from then we kept having phone calls.

Carol, Service user

Likewise, workers appreciated the trust service users placed in them and the generosity shown. For example, workers expressed some surprise that many service users

still rated it as a positive experience despite my misgivings about sort of like, you know, all the hiccups and interruptions and, you know, other headaches of the technology it wrought.

Aran, Worker

Overall, there was an acknowledgment that both workers and service users were learning together and were simultaneously adapting to the impacts of the pandemic and varying forms of lockdown and restriction. These shared experiences appeared to equalize power in some service user–worker relationships.

#### Matching Service Offering With Service User Need

Different forms of telehealth were perceived to be more or less helpful, at different times, for recognizing and responding to varying degrees of distress. All participants acknowledged that telehealth offered a convenience that was not always possible with in-person support. It was handy to quickly and easily obtain scripts for medication, have a brief medical consultation, and gain emotional support:

...picking up the phone isn't as hard as having to go out and drive all the way kind of to the other side of town to go sit in a waiting room and wait to see someone

Christine, Service user

In addition to savings in time and effort, some service users found that the telephone provided a safer space for psychosocial support

because you are not face-to-face with them in person. It feels more like a safer zone that, you know, you don't see that person

Li, Service user

However, for some service users, particularly those experiencing high levels of distress, telephone sessions felt far more challenging and unsafe than in-person support. One person in particular described feeling very alone and unsafe with all forms of telehealth:

Oh, I actually hate it. I was really surprised with hearing how it worked for other people because it just doesn't work for me. It really doesn't feel safe to me and, yeah…..like, when I'm really distressed or going into heavy things or vulnerable things, it's just I just often feel like I can't do it and I can't do it on my own and I need a lot of help and support and I just don't feel the support through telehealth.

Anita, Service user

Service users who found it more challenging to communicate distress over the telephone appreciated the visual cues offered by videoconferencing. This was very important for people who described having trouble expressing themselves when feeling unwell:

I end up losing a lot of language and so if they don't have the visual cue, I feel like possibly they don't actually pick up how not good I actually am.

Mia, Service user

Videoconferencing platforms added an extra challenge for some service users who reported that seeing their own image on screen made them feel uncomfortable and unsafe. For 2 participants with eating disorders, this experience was at times so distressing and distracting they reported needing to end the session earlier than scheduled.

#### Having and Exercising Choice and Control

If I had an option to pick face-to-face or telehealth, I'd probably go face-to-face but if the question was, ”What's easier?“ I'd say telehealth

Carol, Service user

Participants told us that having some capacity to control the terms of engagement with telehealth led to more positive experiences. Although the organization encouraged the use of a specific digital teleconferencing platform, workers found that many service users did not have the equipment or sufficient data required to gain access. In response to these challenges, many workers chose to adopt the platform of the service user’s choice. Workers and service users reported that platform preferences were usually based on familiarity, ease of access, and confidence. In addition to choice in platform type (eg, phone, video, text messaging), workers also offered choice in the timing and availability of connection (eg, when and for how long, planned, short notice, spontaneous, asynchronous) and in the focus of the session (eg, putting some things on hold that did not feel right to focus on via telehealth, choosing matters of highest priority).

Workers told us that by offering choices, they hoped to increase service users’ engagement, safety, and comfort with telehealth. However, the reality was that a significant number of service users did not have real choices or the ability to exercise control. For example, some did not have the technology or the data available to them to engage with videoconferencing or even phone contact—

...they literally don’t have the tools to do it...

Violet, Worker

or could not navigate platforms on their own. Workers were concerned that a significant number of people had dropped out of contact with the service, and although some service users had indicated that they would resume contact when meetings could be in-person, others just disappeared. This seemed to be particularly the case for people engaged with group-based psychosocial interventions:

I saw people just fall away.

Ava, Worker

### Spaces and Strategies That Enable Privacy and Safety

The physical space in which telehealth sessions occurred mattered to both workers and service users. A small number of service users noted that spaces in their homes did not afford the safe “therapeutic holding” they experienced in a physical office with a physically present support worker. This meant that those service users did not engage in the same therapeutic work they might have done had they attended in-person. Irrespective of the nature of the therapeutic work, feeling safe in a telehealth session for most service users meant that those they lived with could not overhear their sessions. Of equal importance for service users was the need to be reassured that their support worker was also engaging with them from a quiet and private place:

There's been times where it's been challenging and I was like, ”Oh, am I actually - do I want to say stuff because I don't want anyone else to hear…..“ I would tell her I don’t feel comfortable saying some things when I can hear other people in the back.

Carol, Service user

Challenges to safety and privacy seemed to be exacerbated in group-based work where both service users and workers had less control over the contexts of the other group participants;

I’ve had people ask me, who is listening to this? Is this being recorded?

Ava, Worker

Notably, for all participants in this study, safety was also associated with the relational space afforded or restricted by telehealth;

I started to realize how much of my job isn't actually about what people say but picking up on other things.

Violet, Worker

Some service users sensed that their support workers had more time available when using telehealth; this created less time pressure during their exchanges, allowed for more conversation, and enhanced a sense of safety. Being able to control the level of anonymity in the exchange was also important for feelings of safety and comfort. This often meant that it was significant for service users to have the choice of turning their video on or off in sessions;

I could also have the option of turning off my camera but still being there, and they were very like accepting of that.

Carol, Service user

Although all workers expressed support and understanding for service users who wanted to only show a blank screen, or be the voice at the end of a phone line, some expressed doubts about their ability to connect when working with very vulnerable people they could not see—

Really hard to do on the phone because there's that whole disconnect.

Ava, Worker

Of note, few workers reported the need to address the security of online platforms used for telehealth with service users. This may be because while the security of the online platform used was important for some service users, most of the people we interviewed stated that they either had not thought about it or were not concerned. For some of the service users, trust in their worker influenced their trust in the platform used for telehealth:

I trust that the people that I'm having the Zoom meeting with have done their homework and they've chosen a platform which is going to be secure and not be hacked…

Laura, Service user

### A Whole New Way of Working

#### Doing Things Differently

The data revealed that telehealth was not just about a different mode of connecting; it required a whole new way of working. Unable to rely on previously proven ways of providing psychosocial support, workers made great efforts to be flexible and responsive in maintaining engagement and meeting people’s needs. Workers told us they needed to think and work flexibly and differently.

While the flexibility of new and different ways of working was appreciated by many service users, for some, the increased flexibility came at the cost of predictability. For one participant, changes to service delivery meant that there was

way too much choice

and this left her feeling as though she

didn't know the rules anymore.

Anita, Service user

Workers told us that the loss of predictability and constant need for flexibility and adaptation left them feeling fatigued. Constantly adapting meant that changes to practice were often made in the moment. Sessions became shorter and sometimes more frequent; poor internet connections were managed by quickly switching to phone; and a lack of privacy or safety at home was accommodated by doing sessions in the car, or at a local park, or at a different time altogether. Most of the workers felt as though they were constantly “winging it”; this was the case even for those with self-described high-level technological skills.

A number of service users told us that despite workers' best efforts, the real value of psychosocial support was lost in telehealth because it did not give them the social interaction and practical support important for their well-being. As one participant said,

I still don’t know how to handle life- for me to do this I need practice.

Hannah, Service user

Workers were particularly concerned that telehealth was not suitable for people experiencing financial hardship, unstable accommodation, homelessness, or in prison:

So, although we've got the platforms available…I think they really need that sort of practical face-to-face engagement, like either center based or outreach.

Liam, Worker

#### Good Practice in Telehealth Takes Time, Effort, and Organizational Support

Although most service users identified time savings as one of the major benefits of telehealth, workers described many aspects of telehealth as taking considerable time and effort. Being responsive to service user needs and working flexibly meant that extra time was required for planning, building technological skills, and developing supportive relationships. Workers spent time providing technical support to service users while simultaneously learning how to use new platforms themselves. They reported variable levels of organizational recognition of the time required to engage via telehealth and expressed a need for

having dedicated time to become proficient

Lucas, Worker

themselves, as well as dedicated time and resources to support service users’ technology needs:

They [service users] just found it too hard which was really disappointing because it's something I was quite excited about and they just found it too hard to do and I guess being able to sort of explain or sort of help someone navigate that process.

Liam, Worker

Workers told us that it was

harder to make that connection...

Violet, Worker

with service users via telehealth, and described feeling drained and

stretched thin

Lucas, Worker

by the extra time and effort needed to build rapport. Interestingly, although most workers felt that a preexisting relationship with a service user should have made the transition to telehealth easier it was not always the case:

Even though we already had a prior relationship, I didn't have that sense from them that, this is a safe space, and I can talk about anything that I need to talk about in this time. They were more guarded, so I was having to work a bit harder.

Ava, Worker

While most research participants expressed a preference for a return to in-person psychosocial support, or hybrid in-person and telehealth models, there was a general recognition that intentional use of telehealth could contribute to flexible and responsive service delivery. Workers were hopeful that offering telehealth in future could enable better access to services for people who often find it more difficult to connect with support such as those in remote areas and people living with disabilities. Workers identified 4 key foundations necessary to build their capacity and confidence to deliver high quality psychosocial supports via telehealth. These were (1) receiving the training they need, (2) having dedicated time to develop their practice and do the behind-the-scenes work required, (3) spending time as a team to work through challenges and discover solutions collectively, and (4) having a supportive and confident manager who championed the use of telehealth. These 4 foundations were particularly important to workers with lower self-reported levels of confidence using technology.

## Discussion

### Principal Findings

We examined the experiences of Australian community mental health service users with the transition to telehealth service delivery during the COVID-19 pandemic. Overall, our findings highlighted that most service users experienced telehealth positively, but this was dependent on them continuing to get the support they needed in a way that was safe and comfortable. The first of 2 themes was ensuring telehealth was a safe means of interacting. Among a population with high rates of prior trauma and past experiences of involuntary or coerced treatment, trust, and safety are especially important in service delivery [16].

In common with previous telehealth implementations [5,7,17], service users indicated a need to adapt to new ways of interacting with services, and workers expressed concerns that technical difficulties and a lack of in-person presence would interfere with maintaining an effective helping relationship. We found that during this pandemic, when telehealth was typically the only way to receive psychosocial support, service users were generally willing to adapt, and adjustment to a new service delivery model could be navigated for most. Generating a sense of safety and ensuring needs could still be met appeared important to successful telehealth service delivery. Notably, safety did not seem to be related to security or usability of the telecommunication platforms used. Rather, this appeared primarily related to being able to maintain valued elements of supportive relationships with workers. However, it appeared that workers could foster this sense of safety and continuity of support by ensuring clients could exercise choices about which communication modalities to use and how to use them. This also allowed for opportunities presented by telehealth to be capitalized upon, such as convenience of access and appointments being less rushed. In line with other reports of the use of telehealth [1-7], using more remote communication channels sometimes overcame discomfort associated with in-person interaction. There also appeared to be a potential to increase the power and control the client had within the relationship through these choices, which may be especially important in this often-disempowered client group.

The service user’s own environment was also a major factor in the creation of a sense of safety, with privacy being raised as an issue, consistent with the findings of some other examinations of telehealth [18,19]. Potentially, this can be navigated by problem solving—finding a suitable location for telehealth calls to take place. However, it is also important to note that workers reported disengagement from a number of service users who were unable to use telehealth and concern in meeting the needs for persons without stable accommodation, who may be most in need. This is in line with international reports of experiences, for example, a major survey of UK mental health practitioners found the majority had lost contact with some service users due to the shift to remote working [12].

The second overall theme highlighted some of the demands that the shift to telehealth resulted in for both service users and workers, including a need for flexibility, and tolerance of unpredictability. Several worker participants cited technological issues experienced by both themselves and service users as a key challenge to providing effective support via telehealth. This emphasizes the importance of services that ensure supporting systems are prioritized at an organizational level in order to allow workers to have the technical support and equipment needed, in good time [20]. In common with previous research findings [7], workers also experienced telehealth as fatiguing. This partly appeared attributable to having to adapt to a new mode of practice, but also to having to meet the additional out-of-session work required and facing greater challenges in reading nonverbal cues to maintain rapport. It is notable that randomized controlled trials have not found formal client ratings of therapeutic rapport to be lower with telehealth than with in-person delivery [5,6,21]. On the other hand, the demand on workers in maintaining rapport can be greater in the absence of nonverbal cues.

Notably, the challenges to telehealth provision of psychosocial support identified in this study, in particular creating the sense of walking alongside people in life, are yet to be fully understood. Unexpectedly, however, the need for different communication approaches also appeared to create a positive change in the way that both workers and service users thought about the nature of service interaction. This appeared to open up the possibility of new ways of practice, by embracing remote communication as a previously underutilized means of connecting with and supporting clients. This aligns with hopes that the rapid introduction of telehealth may create opportunities to transform care [22,23]. Overall, this study highlighted that, for participants, the introduction of telehealth represented a fundamental change in practice than merely a change in means of communication.

Findings highlight a range of enablers and opportunities that can inform service delivery during the immediate postpandemic period and beyond. They demonstrate that more flexible ways of providing support, including hybrid approaches combining face-to-face and telehealth options, are welcome and should be embraced. However, service changes must be accompanied by acknowledgment that telehealth involves a different way of working together and not simply a different platform. Adequate supports—resources; training; coaching and encouragement; and time to practice, build skills, and confidence—are required to enable both service users and workers in navigating the new way of working and the new rules of engagement. Positive early telehealth interactions are critical in supporting people, to persist and overcome any technical issues that arise; therefore, organizations need to foster positive attitudes and skills in staff around the potential and practice of telehealth [24] for psychosocial service delivery. Resistance to more hybrid modes of psychosocial service delivery is likely to persist from some workers and service users; however, witnessing or hearing about the benefits and being adequately supported to adapt to new ways of working may overcome this hesitation. The ongoing use of telehealth in psychosocial interventions and supports will likely see continuing creative adaptations that will benefit those using these services; however, our findings highlight the crucial role of choice and caution against wholly telehealth service provision; most service users did not want this.

### Strengths and Limitations

Our study strengths were the naturalistic examination of telehealth implementation, with a research team combining lived experience and multidisciplinary expertise. However, while the researchers tried to source people who had less positive experiences of telehealth, workers informed us that many of these service users had ceased contact with Neami. Furthermore, the use of telephone or videoconferencing to conduct interviews were barriers to including a sample representing all views, and impressions of these experiences relied upon reports from workers. It should also be noted that experiences of telehealth implementation during the unique circumstances of the COVID-19 pandemic may not be generalizable to other situations. Indeed, the government restrictions on in-person contact were especially strict and prolonged for many participants in this study.

### Conclusions

The findings of this study suggest that the rapid transition to telehealth created the opportunity for a whole new way of thinking about and providing psychosocial support. While access difficulties of a subgroup of service users should not be ignored, most service users and workers were able to adapt to telehealth adoption by focusing on maintaining the relationship and using choice and flexibility to maintain service delivery. Together with opportunities for increasing access, this suggests that within community mental health services, telehealth has value as a new domain of practice.
